# Supplementary material for: Body mass index trajectories and adiposity rebound during the first 6 years in Korean children: Based on the National Health Information Database, 2008–2015
Source: PLoS One. 2020 Oct 30;15(10):e0232810. doi: 10.1371/journal.pone.0232810 (PMC7598489; doi:10.1371/journal.pone.0232810)
Supplement: Attachment — Submitted filename: 20200912 Response to Reviewers comments.docx [file pone.0232810.s001.docx]

**Response to reviewer’s comments**

We appreciate the reviewer’s comments, which have helped us to significantly improve our manuscript.

#1. The purpose/objective of this paper should be refined. There is too much information for a single paper. As you do not have information regarding gestational age of the participants, as the subgroups according to birthweight (VLBW, LBW and non-LBW) are really unbalanced, and as the timing of AR was not different between these subgroups, I suggest you delete them from the paper and only focus on the different types of rebounds, RWG and the factors associating with.

Answer: I agree with your opinion. As your recommendation, we deleted the part of birth weight- specific BMI growth trajectories or subgroup analysis by birth weight in this manuscript.

#2. I suggest you revise the name of your AR groups. What you call “Early AR” (AR before the age of 45 months (3.75 years)) actually meets the criteria of a very early adiposity rebound, and what you call “moderate AR” (AR at 57 months (4.75 years)) actually is the early AR. Even though you justified this point in the discussion section (“Generally, EAR was defined before 5 years of age, however over 75% of subjects had AR before 57 months…”), the data collection was not performed after the age of 6 years, so children exhibiting a late rebound were not considered in the analyses, and, consequently, median age of AR might appear lower than it is. In a previous paper (<https://doi.org/10.3345/kjp.2018.07227>) you reported that in both boys and girls in the 50th percentile of the curve, adiposity rebound occurred at age 66 months (5.5 years). Accordingly, every rebound occurring before this age in your population should be considered and classified as early.
Finally, the “late AR” group should be renamed “moderate-to-late AR”, as a late AR is usually an AR occurring after the age of 7, and the exams don’t go after 6 years of age in your population.
Answer: Thank you for your meticulous review. As your recommendation, we changed terms indicating AR timing as follows: early → very early; moderate → early; and late → moderate-to-late.

#3. During the 2nd part of the introduction, the authors insisted on SGA children but the authors do not have the information regarding gestational age of their participants. In line with my first comment, please revise this part.
Answer: Yes, we agree with your comment. Rapid catch-up growth in low birth weight infants, who were included in our study, was the focus of the 2nd paragraph of the introduction. Therefore, we revised this paragraph to use the accurate term and added relevant references as follows.

#4. In Table 1, it is stated than 100% of the participants reached the AR by 69 months of age, whereas, in the methods section, it is stated that participants classified as “late AR” where those who had “no AR until 69 months of age”. When looking at the BMI curve, it seems that there is no AR for participants in the “Late AR group”. Please, clarify this point. If all of the participants in this group did not reach the AR by 69 months, you cannot present in Table 1 than 100% of the participants experienced the AR by 69 months.
Answer: Thank you for your comment. Expression of ‘100%’ at the last exam point may make confuse as if all subjects reached to AR before 69 months. We changed the prevalence rate of AR at each time point rather than cumulative values.

#5. Why did you not use the usual definition of rapid weight gain? (increase of more than 0.67 in weight-for-age z-score from birth to 6 months)
Answer: Unfortunately, an individual’s data of birth date or exam date is not provided in the National Health Information Database (NHID). That’s why we used median age at each exam in this study. We could not calculate weight-for-age z-score for each time point. So, we arbitrarily defined the criteria of rapid weight gain in this study (5.1 kg), which is the approximately 0.67 changes of weight z-score for the male Korean standard who was born at median birth weight (at birth 3.3 kg, z-score = 0 and at 5 months 8.4 kg, z-score = 0.67) and this also corresponds to 5-month-changes (birth to 5 months) for the 99^th^ percentile of the male Korean standard. We revised this method section to express them more clearly.

#6. Methods; please, elaborate regarding the questions asked/questionnaires regarding lifestyle habits performed and when they were performed, because we only discover this information in the results section.
Answer: We added detailed descriptions about questionnaires included in Health check-ups for infants.

\# 7. Statistics section; Please, add the modeling of the BMI percentile curves.
Answer: We used a mixed effects linear model which covers the individual’s longitudinal data characteristics (unequal space/intervals, and missing value). We added this description in Data analysis section.

#8. Results, section “BMI trajectories during the first 6 years”
In this section, you compare the BMI values at each time points between girls and boys, and these values were different at each point. However, it does not necessary mean that boys and girls do have a different BMI trajectory. Please, can you specify how you define “BMI trajectory” and how you statistically found that those trajectories are different between boys and girls?
Answer: We simply compared BMI values at each time points between males and females by student’s t-test. While comparison of BMI trajectory which implies the BMI changes over time between males and females were analyzed using a mixed effect linear model. We added this description in Data analysis section.

\# 9. Still in the “BMI trajectories during the first 6 years” results section; you say that 10,209 subjects were in the EAR group, 2,697 in the moderate AR group and 3,301 in the “late AR group”. So there is, in total, 16,207 subjects. However, in table 2, where you show participants with BMI values at age 57 months (exam 6) you only have 11,106 subjects left, but your Figure 1 (flow chart) shows that, at Exam 6, you have 17,590 participants. Please, explain why you lost so many participants (which constitute almost the half of your EAR group).
Answer: The reason why there exist differences of number of subjects (AR timing analysis: 16,207 subjects; Exam-VI and AR timing analysis: 11,106 subjects) is we included an individual’s longitudinal data which have at least five valid height and weight measurements from seven health check-ups. This means that subjects may have one or two missing data. If this missing data does not affect to AR timing analysis, it could be included in 16,207 subjects. But if this missing data is the data of Exam-VI, it could not be included in 11,106 subjects. For example, ‘A’ subject has early AR before Exam-IV, but does not have BMI data of Exam-VI, then ‘A’ subject is excluded from Table 2. Therefore, differences of number of subjects were bigger in ‘Very Early’ AR group than ‘Early’ or ‘Mod-to-Late’ groups.

#10. Results, section “The timing of adiposity rebound”.
I do not understand the following sentence “The median and mode of the timing of AR was 45 months (Exam-V) and 33 months (Exam IV) of age, respectively, in all subjects”. Please, clarify or correct.
Answer: The mode value of AR timing does not imply clinical significance, so we erased this part.

#11. Table 2: Instead of investigating the OR for adiposity rebound depending on body weight status, I suggest you rather investigate the OR for underweight, overweight or obesity depending on the timing of the AR, as the AR is known as being a risk factor of obesity. Then, you could adjust your regression on various factors such as breastfeeding, BMI of the parents, screen use, sugar-sweetened beverage intake at 21 months, exercise habits etc..
Answer: Thank you for your valuable opinion. The aim of this study was to find related factor to early AR, therefore, we focused to outcome (early AR) in the analysis. Your suggestion is also very interesting, but unfortunately, data in NHID is not freely accessible at this moment so we could not investigate the OR for BMI status depending on the AR timing. Instead, we added showing percentage according to the BMI status at age 57 months.

#12. Table 3: Instead of showing percentage according to the dependent variable (EAR, moderate or late AR), you should show them according to the independent variable, between the different groups (e.g Among all breastfeed children, please show the proportion of them being in the EAR, the Moderate and Late AR groups).
Answer: Thank you for your comment. We changed showing percentage according to the independent variable.

#13. The manuscript should be revised by an English-native person, especially the results section which is unclear and clumsy.
Answer: Initial manuscript had undergone English proofreading by the editage (<https://www.editage.com>). Revised final manuscript will be undergone English proofreading again before publishing.

#14. Just a thought: among your participants with “EAR” (45 months), about 25% will be overweight/obese by 5 years old. It also means than 75% of them should not experience weight issue (at least at 5 years of age). This maybe suggests that the classic way to define the adiposity rebound is not discriminant enough and calls for a new approach/definition in order to target children at risk for later obesity.

Answer: Thank you for your bright idea. I will keep this in mind.
